# Comparison of Child-Pugh and MELD scores in predicting survival of hepatocellular carcinoma patients with splenomegaly undergoing TACE: a real-world study

**DOI:** 10.3389/fmed.2026.1804646

**Published:** 2026-06-10

**Authors:** Lu Xu, Bo Jiang, Yicheng Ma, Jianhua Pan, Ming Liang, Qianqian Du, Haotian Wu, Zhiheng Cheng, Dong Lu

**Affiliations:** Invasive Technology Department, Second Hospital of Anhui Medical University, Hefei, China

**Keywords:** Child-Pugh score, hepatocellular carcinoma, model for end-stage liver disease, prognosis, splenomegaly, transarterial chemoembolization

## Abstract

**Objective:**

This study aims to compare the predictive value of Child-Pugh and MELD scores for the survival rate of HCC patients with splenomegaly who received TACE.

**Methods:**

From January 2018 to January 2025, 306 patients with splenomegaly and HCC who underwent TACE treatment at a tertiary hospital were included in the study. Patients were divided into groups A (*n* = 183), B (*n* = 92), and C (*n* = 31) based on the baseline Child-Pugh score. At the same time, patients were divided into low-risk (MELD<10, *n* = 122), intermediate-risk (MELD10-20, *n* = 122), and high-risk (MELD>20, *n* = 62) groups based on the baseline MELD score. Baseline clinical pathological characteristics were collected. The primary endpoint was overall survival (OS) and progression-free survival (PFS). Survival analysis was performed using the Kaplan–Meier method, and the time series test was used to evaluate the comparison between groups. The receiver operating characteristic (ROC) curve was used to evaluate the predictive performance of the two scoring systems for 1-year postoperative survival rate, and the DeLong test was used for comparison.

**Results:**

The comparison analysis of baseline characteristics showed that there were statistically significant differences in age, maximum tumor diameter, proportion of multifocal tumors, spleen length, total bilirubin, albumin, international normalized ratio (INR), creatinine, red blood cell distribution width (RDW), platelet count, and the incidence of ascites and hepatic encephalopathy among different Child-Pugh grades (all *p* < 0.05). Similarly, significant differences were observed in the above parameters among different MELD risk groups (all *p* < 0.05). Survival analysis indicated that with the increase in Child-Pugh grade, OS and PFS were significantly decreased (log-rank χ^2^ = 45.332, *p* < 0.001; χ^2^ = 38.775, *p* < 0.001). Similarly, with the increase in MELD risk category, OS and PFS significantly decreased (log-rank χ^2^ = 52.114, *p* < 0.001; χ^2^ = 41.889, *p* < 0.001). The predictive performance analysis indicated that compared with the Child-Pugh score, the MELD score had a significantly larger area under the curve (AUC) for predicting 1-year survival rate (DeLong test Z = 3.124, *p* = 0.002).

**Conclusion:**

In patients with HCC and splenomegaly, both Child-Pugh and MELD scores can effectively stratify prognosis. However, the MELD score showed superior predictive performance for 1-year survival rate after TACE, which is worthy of more attention in clinical prognosis assessment.

## 1 Introduction

Hepatocellular carcinoma (HCC) is one of the leading causes of cancer-related morbidity and mortality worldwide, and its treatment strategies largely depend on liver functional reserve and tumor stage (1). Transarterial chemoembolization (TACE) is a widely used and guideline-recommended standard palliative treatment for patients with inoperable advanced hepatocellular carcinoma (2). However, patients with HCC often have cirrhosis and portal hypertension, leading to splenomegaly. These patients have a more complex clinicopathological profile, characterized by further impairment of liver functional reserve and a more pronounced systemic inflammatory state, which together affect tolerance to TACE and long-term survival outcomes (3, 4). Therefore, accurate prognostic assessment of HCC patients with splenomegaly is crucial for making personalized treatment decisions (5). In clinical practice, the Child-Pugh classification and the Model for End-Stage Liver Disease (MELD) score are the two most commonly used tools to assess the severity and prognosis of liver disease (6, 7). The Child-Pugh classification combines clinical symptoms such as ascites and hepatic encephalopathy with laboratory parameters, has a long history of use, and is simple to operate in clinical settings (8). In contrast, the MELD score is derived from three objective variables—creatinine, bilirubin, and the international normalized ratio (INR)—and is designed to more objectively quantify the risk of death from end-stage liver disease. Originally developed for liver transplant allocation, its application has been extended to prognostic prediction in various liver disease settings (9, 10).

Although the prognostic value of the Child-Pugh and MELD scores has been widely validated in the general cirrhosis or HCC population, comparative analysis of their predictive effects in specific HCC subgroups with splenomegaly remains controversial and limited (11). First, splenomegaly is a direct consequence of portal hypertension and is often associated with high output circulation, hypersplenism, and more severe liver fibrosis. Traditional Child-Pugh or MELD scores may not fully capture these factors (12). Although albumin and bilirubin in the Child-Pugh classification reflect synthetic and excretory liver function, they are insufficient to describe the severity of portal hypertension itself and the subsequent systemic pathophysiological changes (13). Secondly, although the MELD score provides objectivity, its core variables mainly reflect renal and liver excretory functions, lacking direct assessment of liver synthetic function (such as albumin) and complications of portal hypertension (such as ascites, degree of splenomegaly) (14). Most existing studies comparing the prognostic value of these two scores in HCC patients only analyze splenomegaly as one of other confounding factors or do not specifically explore in-depth in the cohort of splenomegalic patients receiving TACE. This leads to inconsistent conclusions and the scarcity of direct comparative evidence from large-sample, real-world data.

In this context, this study aims to retrospectively analyze the clinical data of a real clinical cohort of HCC patients with splenomegaly who received TACE treatment. It attempted to systematically compare the ability of baseline Child-Pugh classification and MELD score to stratify postoperative overall survival (OS) and progression-free survival (PFS). In addition, it evaluated the predictive effect of these two scores on short-term (e.g., 1 year after surgery) survival. The results of this study are expected to provide more precise evidence-based guidance for personalized prognostic assessment and optimization of clinical management strategies for HCC patients with splenomegaly.

## 2 Materials and methods

### 2.1 General data

This single-center retrospective cohort study included patients with hepatocellular carcinoma (HCC) and splenomegaly treated at a tertiary care hospital from January 2018 to January 2025. All patients underwent transarterial chemoembolization (TACE). The total sample size was 306 patients. Sample size was calculated based on expected effect size and statistical power: according to literature reports, the expected difference in area under the survival curve (AUC) between Child-Pugh and MELD scores is approximately 0.1–0.2 (13). When *α* = 0.05 and *β* = 0.2 (power = 80%), the minimum required sample size calculated using PASS software was 300 patients; 306 were finally included to enhance robustness. According to Child-Pugh classification, patients were divided into three groups: approximately 60% were Child-Pugh A group (183 patients), approximately 30% were Child-Pugh B group (92 patients), and approximately 10% were Child-Pugh C group (31 patients). Meanwhile, patients were stratified according to MELD score: low risk (MELD<10) (*n* = 122), intermediate risk (MELD10-20) (*n* = 122), and high risk (MELED>20) (t = 62) to cover the full spectrum of liver disease severity. Baseline patient characteristics included age, sex, etiology (e.g., hepatitis B, alcoholic liver disease), tumor characteristics (size, number), and spleen size (measured by imaging).

### 2.2 Inclusion and exclusion criteria

Inclusion criteria:

(1) Age ≥ 18 years;
(2) HCC diagnosis confirmed by imaging criteria (such as enhancement in the arterial phase accompanied by portal vein or delayed phase perfusion) or through pathological biopsy, and confirmed by computed tomography (CT) or magnetic resonance imaging (MRI);
(3) Presence of splenomegaly, defined by CT or MRI as spleen length greater than 12 cm, spleen thickness greater than 4 cm, or maximum diameter of the splenic hilum greater than 5 cm, or clinical palpation showing that the lower pole of the spleen extends beyond the left costal margin (15);
(4) All patients received at least one TACE treatment and had complete treatment records;
(5) Complete clinical data available, including laboratory parameters required for calculating Child-Pugh and MELD scores: total bilirubin (unit: μmol/L), albumin (unit: g/L), international normalized ratio (INR), creatinine (unit: μmol/L), as well as records for evaluating ascites and hepatic encephalopathy.

Exclusion criteria:

(1) Comorbidity with other malignant tumors, such as cholangiocarcinoma or metastatic liver cancer, to avoid confusion in survival analysis;
(2) Preoperative enhanced CT or MRI assessment revealing major vascular invasion (involvement of the main portal vein [Vp3/Vp4], hepatic vein, or inferior vena cava) or distant metastasis (extrahepatic metastasis, such as lung, bone, lymph nodes), as these patients typically have a poor prognosis and are not considered ideal candidates for TACE according to clinical guidelines;
(3) Prior liver transplantation or absolute contraindications to TACE, such as severe cardiac dysfunction (New York Heart Association class III-IV), renal insufficiency (creatinine clearance rate <30 mL/min), or contrast agent allergy;
(4) Lack of key data, inability to calculate Child-Pugh or MELD scores, or incomplete follow-up information;
(5) Follow-up for less than 6 months to ensure the reliability of survival data;
(6) Pregnancy or lactation;
(7) Concurrent severe infection (such as sepsis requiring antibiotic treatment) or active autoimmune disease (such as systemic lupus erythematosus requiring immunosuppressive therapy).

### 2.3 Equipment and materials

(1) Imaging equipment: multi-detector spiral CT scanners or 3.0 Tesla (T) MRI systems are used for abdominal imaging to measure the size of the spleen and tumor characteristics. The scanning parameters follow the standard hospital protocols: slice thickness of 5 mm; enhanced scans use iodine-based contrast agents for CT or gadolinium-based contrast agents for MRI. The examination is conducted and interpreted by experienced radiologists to ensure consistency in measurements. The size of the spleen is measured on axial images and the average of three measurements is taken.
(2) Laboratory equipment: a fully automatic biochemical analyzer (Siemens ADVIA Chemistry XPT) is used to detect serum total bilirubin, albumin, creatinine, and other indicators. All tests followed international standardized operating procedures (SOP) and underwent daily internal quality control. A hematology analyzer (Mindray BC-7500 CS) was used to measure red cell distribution width (RDW, expressed as a percentage) and platelet count. Coagulation function, especially the international normalized ratio (INR) of prothrombin time, was measured using a coagulation analyzer (Sysmex CS 5100). Laboratory values were taken from the most recent test results obtained before baseline treatment.
(3) TACE treatment equipment: TACE procedures were performed using the Progreat microcatheter system (Terumo Corporation) and contrast agents (such as iodized oil) under the guidance of digital subtraction angiography (DSA) machines (such as Siemens Artis series). All equipment was part of the hospital’s standard configuration. Interventional radiologists performed procedures according to standardized institutional protocols, including percutaneous femoral artery puncture and hepatic artery branch superselective catheterization. Conventional TACE (cTACE) was performed with an emulsion of iodized oil (lipiodol) and chemotherapy drugs (such as pirarubicin, epirubicin, lobaplatin), followed by embolization with particles (such as gelatin sponge, PVA particles). Drug-eluting bead TACE (DEB-TACE) was performed using CalliSpheres microspheres loaded with chemotherapy drugs (such as epirubicin). The selection of protocols (cTACE vs. DEB-TACE) and specific drugs was determined by interventional radiologists based on tumor characteristics (size, vascularity) and patient performance status. For this analysis, survival was calculated starting from the first TACE treatment. Equipment maintenance and calibration were performed regularly to ensure treatment safety and data reproducibility.

### 2.4 Research methods

(1) Research design: a single-center retrospective cohort design was adopted. Data were extracted from the Hospital Information System (HIS) and Picture Archiving and Communication System (PACS). The data collection period is from January 2018 to January 2025. Follow-up will be until December 2025 to ensure that all patients have a follow-up period of at least 6 months. Follow-up was conducted through outpatient records, telephone interviews, or death registration systems.
(2) Data collection: two researchers (both with medical backgrounds) independently extracted data using a blinded method, which means that the extractors were unaware of the study group allocation to reduce bias. Extracted content included demographic data (age, gender), etiology (e.g., HBV infection status), laboratory values (total bilirubin, albumin, INR, creatinine, RDW), imaging characteristics (spleen size, maximum tumor diameter, number of tumors), and treatment details (TACE date, drugs used, embolization materials). Data is entered into a custom Excel template and logically validated.
(3) Grouping and variable definition: patients were grouped according to the Child-Pugh score and MELD score as described in Section 2.1. In addition, subgroup analysis was performed to explore heterogeneity, such as dividing patients into two groups based on the median spleen size (13 cm) (“spleen enlargement ≤13 cm” and “spleen enlargement >13 cm”) or the main etiology (HBV-related and alcohol-related). Other variables, such as red blood cell distribution width (RDW), were introduced and analyzed as continuous variables because the literature suggests that they are associated with liver disease prognosis. All variable definitions were based on clinical guidelines and no custom scales were used.
(4) Quality control: a strict quality control process has been implemented. All data were entered in duplicate; discrepancies were adjudicated by a third senior hepatologist. Data audits are conducted monthly, and 10% of cases are randomly selected to recheck key variables (such as laboratory values and imaging measurements), with the error rate controlled below 2%. Audit results are recorded and used to continuously improve the data collection process.
(5) Reasons for TACE in pediatric Pugh class C patients: in this real-world cohort, 31 patients with Child-Pugh class C liver function underwent TACE. These patients represent a strictly selected subgroup and meet all strict institutional criteria: (i) Eastern Cooperative Oncology Group (ECOG) performance status ≤2; (ii) no major vascular invasion (major portal vein, hepatic vein, or inferior vena cava) or extrahepatic metastases; (iii) Child-Pugh score 10–1 1 (i.e., grade C, no grade IIIIV hepatic encephalopathy or refractory ascites); (iv) tumor burden does not exceed the “less than grade 7” criteria; and (v) no other effective treatment options (such as intolerance to sorafenib/levetiracetam, financial constraints, or inability to receive systemic therapy during the 2018–2021 treatment period). This decision was made by a multidisciplinary team (MDT) consisting of interventional radiologists, hepatologists, and oncologists after thorough discussion of risks and benefits, and an informed consent specifically for higher surgical risks was documented. Perioperative risk management included: (a) preoperative optimization of albumin infusion (target serum albumin > 28 g/L), diuretics for ascites control, lactulose for subclinical encephalopathy; (b) use of super-selective segmental/segmental TACE to reduce drug/embolic dose (iodized oil ≤ 6 mL, chemotherapy drugs at 50% of the standard dose); (c) intraoperative monitoring of vital signs and central venous pressure; (d) 24-48-h postoperative observation in the intensive care unit (ICU). And (e) prophylactic antibiotics and liver protectants (such as aspartate ornithine, branched-chain amino acids). Despite taking these measures, we acknowledge that the risk for Child-Pugh C patients undergoing TACE is very high. Our results should not be interpreted as supporting routine TACE for this population.

### 2.5 Outcome measures

(1) Overall survival (OS): Defined as the time (in months) from the date of TACE to the date of death or the last follow-up date. Death information was verified through hospital death records, family confirmation, or the national death registration system. OS was the primary endpoint, described visually using Kaplan–Meier survival curves.
(2) Progression-free survival (PFS): Defined as the time (in months) from the date of TACE to tumor progression (according to the modified Response Evaluation Criteria in Solid Tumors [mRECIST]) or death. Tumor progression was evaluated by CT or MRI imaging, independently assessed by two radiologists who were blinded to the study group; disagreements were resolved through a consensus meeting. According to mRECIST criteria, progression was defined as an increase of ≥ 20% in the sum of diameters of target lesions or the appearance of new lesions.
(3) Child-Pugh score: Baseline score was calculated. This score is based on five parameters: total bilirubin (<34 μmol/L = 1 point, 34–51 μmol/L = 2 points, >51 μmol/L = 3 points), albumin (>35 g/L = 1 point, 28–35 g/L = 2 points, <28 g/L = 3 points), international normalized ratio (INR) (<1.7 = 1 point, 1.7–2.3 = 2 points, >2.3 = 3 points), ascites (absent = 1 point, mild = 2 points, moderate–severe = 3 points), and hepatic encephalopathy (none = 1 point, grade I-II = 2 points, grade III-IV = 3 points). The total score ranges from 5 to 15, categorized as class A (5–6 points), class B (7–9 points), and class C (10–15 points). Scores were assessed by the attending physician prior to TACE.
(4) MELD score: Baseline score was calculated using the standard formula: MELD = 3.78 × ln(total bilirubin, mg/dL) + 11.2 × ln(INR) + 9.57 × ln(creatinine, mg/dL) + 6.43 (where ln is the natural logarithm). Importantly, all raw total bilirubin values (reported in μmol/L) and creatinine values (reported in μmol/L) were first uniformly converted to mg/dL using standard conversion factors (bilirubin: 1 mg/dL = 17.1 μmol/L; creatinine: 1 mg/dL = 88.4 μmol/L). Use a proven spreadsheet macro to calculate each patient’s value before entering the MELD formula. Scores are rounded to the nearest whole number, usually between 6 and 40, with higher scores indicating greater severity of liver disease. Calculations were automatically performed by the laboratory information system and manually verified by two independent researchers to ensure accuracy.
(5) Red blood cell distribution width (RDW): Use a hematology analyzer to measure the baseline RDW value (expressed as a percentage). The normal reference range is 11.5–14.5%; values greater than 14.5% are defined as abnormal. The detection method involves collecting venous blood in EDTA anticoagulant tubes and analyzing it within 2 h of collection to ensure accuracy. RDW was used as a continuous variable for correlation analysis.

### 2.6 Statistical methods

All statistical analyses were conducted using SPSS software (version 26.0, IBM Corp.) or R software (version 4.2.0). The significance level was set at *p* < 0.05 (two-tailed). First, the Shapiro–Wilk test was used to assess the normality of continuous variables. Normally distributed variables were expressed as mean ± standard deviation (SD); non-normally distributed variables were expressed as median (interquartile range, IQR). Categorical variables were expressed as frequency (percentage). Between-group comparisons: For normally distributed continuous variables, the independent samples t-test was used; for non-normally distributed variables, the Mann–Whitney U test (two groups) or Kruskal-Wallis test (multiple groups) was used; for categorical variables, the chi-square test or Fisher’s exact test (when expected count <5) was used. The Kaplan–Meier method was used to generate survival curves for survival analysis, and the log-rank test was used for between-group comparisons. Predictive performance was compared by calculating the area under the receiver operating characteristic (ROC) curve (AUC). The DeLong test was used to evaluate the difference between Child-Pugh and MELD scores in predicting 1-year survival. To construct a combined model, we performed a binary logistic regression analysis with 1-year survival status (survival coded as 0 and death coded as 1) as the dependent variable. Child-Pugh score and MELD score were used as continuous independent variables in the model to maintain the complete granularity of the data. The predicted probability of 1-year mortality for each patient generated by the logistic regression model was then used as a new predictive signature. The discriminative performance of the combined model was evaluated by plotting a ROC curve based on these predicted probabilities and calculating its AUC. This approach allows two scores to be combined without imposing arbitrary cutoff values. The equation of the combined model is: Logit (P) = −4.821 + 0.312 × Child-Pugh score + 0.154 × MELD score. The predicted probability of death was then calculated as P = exp.(Logit(P))/(1 + exp.(Logit(P))). Although we acknowledged the overlapping components of the Child-Pugh and MELD scores (bilirubin and INR), we explored a joint model to determine whether the unique clinical elements of the Child-Pugh score (ascites, hepatic encephalopathy, albumin) could provide incremental prognostic value beyond the objective laboratory parameters of the MELD score. Collinearity was assessed by calculating the variance inflation factor (VIF) for the two predictors in the logistic regression model; the result was 3.1, indicating a moderate but acceptable level of collinearity. For the 1-year survival ROC analysis, the binary outcome was defined as death from any cause within 365 days after TACE. Patients whose last follow-up date exceeded 365 days were considered event-free (coded as 0). Patients who died within 365 days were considered to have experienced the event (coded as 1). Patients who were followed up for less than 365 days and were alive at the last follow-up were excluded from this specific ROC analysis to avoid misclassification. To further evaluate prognostic performance in a time-dependent manner, we also performed time-dependent ROC analysis for 1-year, 2-year, and 3-year survival using the “survivalROC” package in R, confirming the main findings (data not shown). The calibration curve is used to evaluate the calibration degree, and the Hosmer Lemeshow goodness of fit test is used to assess the consistency between the predicted probability and the actual observed outcome. At the same time, conduct decision curve analysis (DCA) to quantify net benefits.

## 3 Results

### 3.1 Comparison of baseline characteristics by Child-Pugh groups

**Figure 1:**
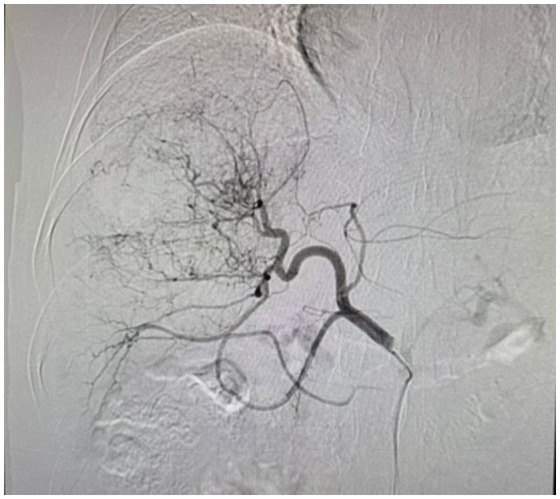
Digital subtraction angiography (DSA) image of a patient with Child-Pugh grade. A 57-year-old man was admitted to the hospital with a 5-day history of upper abdominal pain and discomfort. Imaging examination revealed a large intrahepatic mass, suggestive of primary hepatocellular carcinoma (HCC), accompanied by cirrhosis and splenomegaly. On August 30, 2022, the serum alpha-ferritin (AFP) level was 20547.50 ng/mL, and the Chinese Cancer (CNLC) staging system classified the disease into stage IIb. Transarterial chemoembolization (TACE) was performed on September 1, 2022. During the procedure, 18 mL of iodized oil emulsion (prepared from 20 mL of iodized oil and 40 mg of pirarubicin [THP]) was injected, followed by supplementary embolization using polyvinyl alcohol (PVA) particles with a diameter range of 350–560 μm.

**Figure 2:**
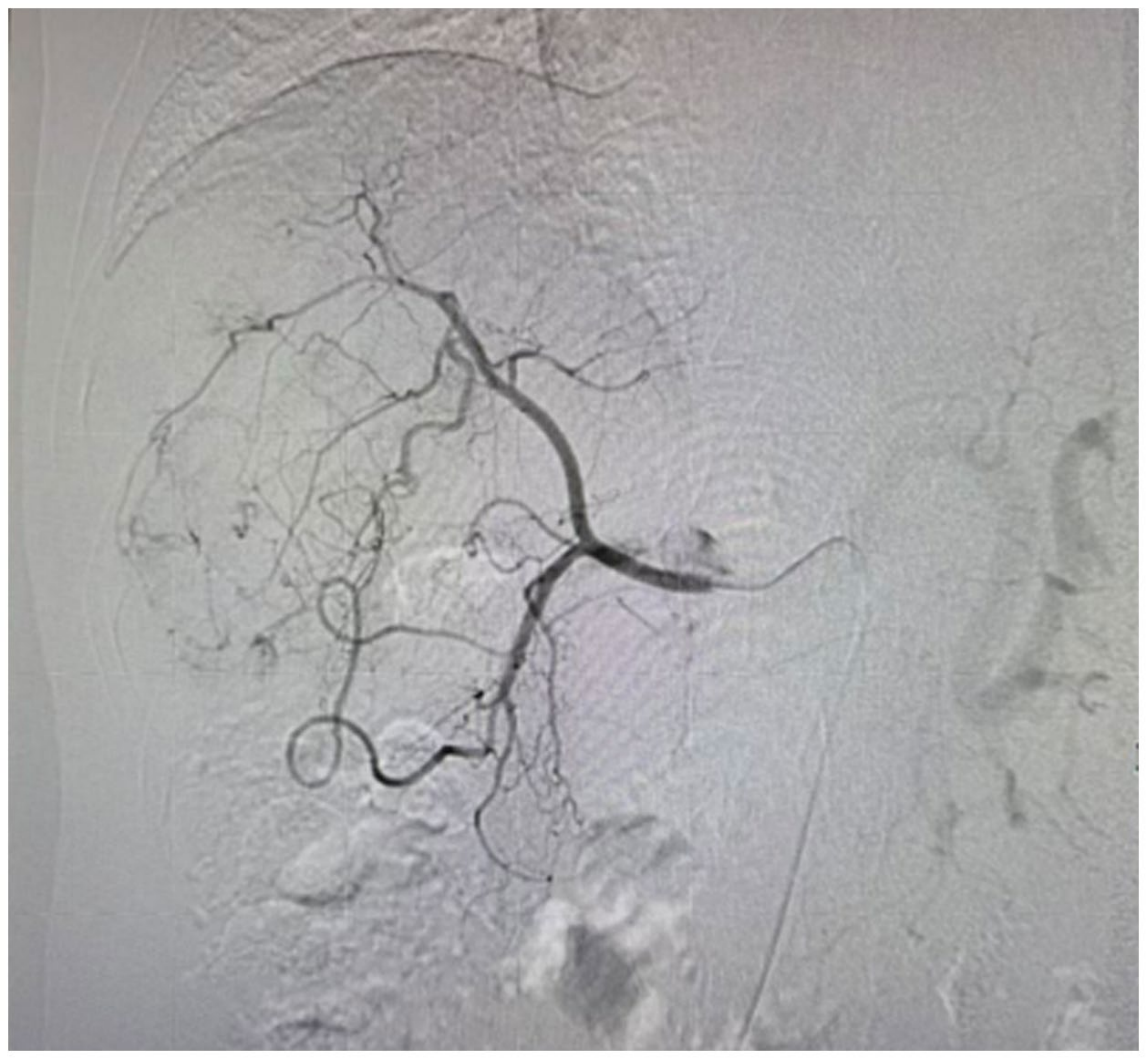
Digital subtraction angiography (DSA) image of a Child-Pugh class B patient. A 69-year-old male with a 6-month history of abdominal distension and potential cirrhosis. Hepatocellular carcinoma (HCC) is located in the lower segment of the right posterior lobe of the liver, with tumor thrombus involving the right posterior branch of the portal vein. TACE was conducted on July 3, 2020. The intervention included the use of 8 mL of iodized oil emulsion (consisting of 10 mL of iodized oil, 40 mg of THP, and 40 mg of cisplatin), and reinforcement with PVA particle (diameter 350–560 μm) embolization.

**Figure 3:**
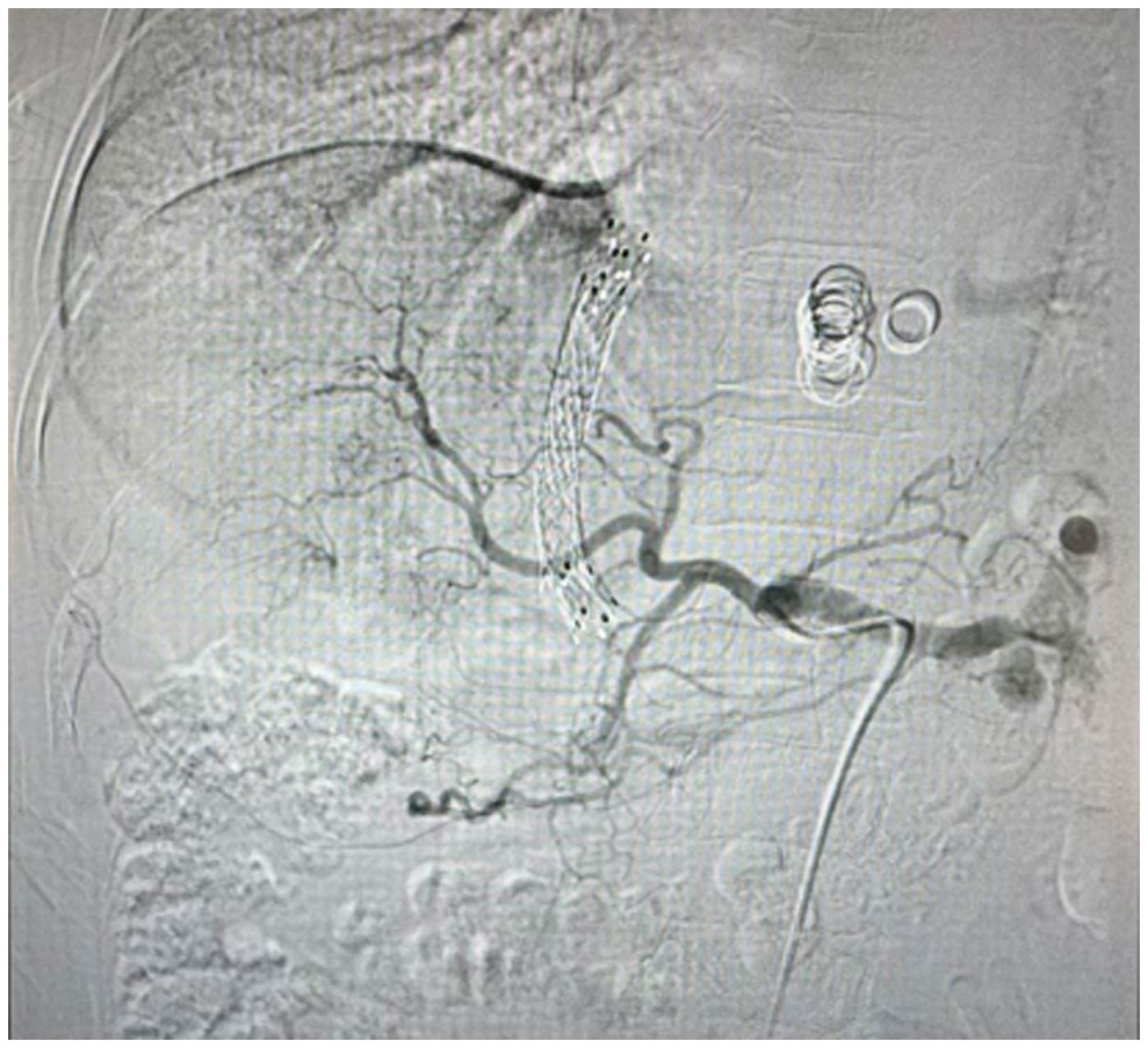
Digital subtraction angiography (DSA) image of a Child-Pugh class C patient. A 55-year-old female was diagnosed with cirrhosis and splenomegaly, accompanied by thrombosis in the main portal vein, superior mesenteric vein, and splenic vein. On September 23, 2021, drug-eluting bead TACE (DEB-TACE) was performed. Angiographic evaluation showed multiple areas of hypervascular staining in the right hepatic lobe. 5 mL of CalliSpheres drug-eluting microsphere suspension was injected, prepared using a vial of CalliSpheres microspheres (size 100–300 μm) loaded with 40 mg of epirubicin and mixed with 5 mL of contrast agent.

According to the comparative analysis of baseline characteristics based on Child-Pugh classification, there were statistically significant differences among the three groups in terms of age, etiology distribution, maximum tumor diameter, proportion of multifocal tumors, splenic length, total bilirubin, albumin, international normalized ratio (INR), creatinine, red cell distribution width (RDW), platelet count, presence of ascites, hepatic encephalopathy, and MELD score (all *p* < 0.05). Specifically, as Child-Pugh class progressed from A to C, patient age, tumor burden indicators, splenic length, total bilirubin, INR, creatinine, RDW, severity of ascites and hepatic encephalopathy, and MELD score increased accordingly. Conversely, albumin levels and platelet counts gradually decreased. There was no statistically significant difference in sex distribution among the three groups (*p* > 0.05) (see Table 1 and Figures 1–3 for details).

**Table 1:** Comparison of baseline characteristics by Child-Pugh groups.

Characteristic	Child-Pugh A (*n* = 183)	Child-Pugh B (*n* = 92)	Child-Pugh C (*n* = 31)	Statistic	*p*-value
Age (years)	51.78 ± 6.92	67.03 ± 2.89	76.03 ± 2.17	*F* = 385.226	<0.001
Male, *n* (%)	152(83.1)	69 (75.0)	24 (77.4)	χ^2^ = 2.643	0.266
Etiology, *n* (%)				χ^2^ = 5.730	0.22
Hepatitis B virus (HBV)	153 (83.6)	69 (75.0)	21 (67.7)		
Alcoholic liver disease	25 (13.7)	19 (20.7)	8 (25.8)		
Other	5 (2.7)	4 (4.3)	2 (6.5)		
Max. tumor diameter (cm), M(IQR)	4.5 (3.1–6.2)	5.8 (4.0–7.5)	6.9 (5.3–9.1)	*F* = 17.672	<0.001
Multifocal tumor (≥2), *n* (%)	67 (36.6)	48 (52.2)	23 (74.2)	χ^2^ = 17.782	<0.001
Splenic length (cm)	13.21 ± 2.34	14.78 ± 2.67	16.05 ± 3.12	*F* = 23.472	<0.001
Total bilirubin (μmol/L), M(IQR)	18.6 (14.2–24.3)	42.5 (35.8–53.1)	78.9 (62.4–95.7)	*F* = 388.3	<0.001
Albumin (g/L)	38.52 ± 4.21	31.76 ± 3.85	26.43 ± 4.02	*F* = 164.36	<0.001
INR, M(IQR)	1.12 (1.05–1.21)	1.52 (1.41–1.68)	1.96 (1.77–2.22)	*F* = 376.6	<0.001
Creatinine (μmol/L), M(IQR)	72.3 (65.1–80.5)	78.9 (69.4–92.1)	101.5 (86.7–124.8)	*F* = 47.46	<0.001
RDW (%)	14.02 ± 1.25	15.67 ± 1.54	16.82 ± 1.75	*F* = 78.67	<0.001
Platelet count (×10^9^/L)	98.34 ± 45.21	76.89 ± 38.47	62.15 ± 31.26	*F* = 14.59	<0.001
Ascites, *n* (%)				χ^2^ = 299.47	<0.001
None	183 (100.0)	30 (32.6)	0 (0.0)		
Mild	0 (0.0)	52 (56.5)	8 (25.8)		
Moderate–severe	0 (0.0)	10 (10.9)	23 (74.2)		
Hepatic encephalopathy, *n* (%)				χ^2^ = 78.51	<0.001
None	183 (100.0)	87 (94.6)	19 (61.3)		
Grade I-II	0 (0.0)	5 (5.4)	10 (32.3)		
Grade III-IV	0 (0.0)	0 (0.0)	2 (6.5)		
MELD score, M(IQR)	8 (7–10)	15 (13–18)	24 (21–28)	*F* = 420.3	<0.001
cTACE/DEB-TACE [cTACE, *n*(%)]	142 (77.6)	68 (73.9)	21 (67.7)	χ^2^ = 1.565	0.457

### 3.2 Comparison of baseline characteristics by MELD groups

Patients were stratified into low-risk (MELD <10, *n* = 122), intermediate-risk (MELD 10–20, *n* = 122), and high-risk (MELD >20, *n* = 62) groups based on baseline MELD scores. The results showed that in terms of age, etiology distribution, maximum tumor diameter, proportion of multifocal tumors, splenic length, total bilirubin, albumin, INR, creatinine, RDW, platelet count, Child-Pugh class, and incidence of ascites and hepatic encephalopathy, there were statistically significant differences (all *p* < 0.05). Trend analysis showed that as the MELD risk category increased, patient age, tumor burden, splenic length, total bilirubin, INR, creatinine, RDW, and the incidence of ascites and hepatic encephalopathy increased correspondingly, while albumin levels and platelet counts decreased, and Child-Pugh class deteriorated. There was no significant difference in sex distribution among the groups (*p* > 0.05) (see Table 2).

**Table 2:** Comparison of baseline characteristics by MELD groups.

Characteristic	MELD>20 (high-risk, *n* = 62)	MELD 10–20 (int.-risk, *n* = 122)	MELD<10 (low-risk, *n* = 122)	Statistic	*p*-value
Age (years)	69.05 ± 6.41	60.42 ± 10.30	52.03 ± 7.14	*F* = 87.508	<0.001
Male, *n* (%)	49 (79.0)	98 (80.3)	98(80.3)	χ^2^ = 0.052	0.974
Etiology, *n* (%)				χ^2^ = 15.234	0.004
Hepatitis B virus (HBV)	40 (64.5)	96 (78.7)	107 (87.7)		
Alcoholic liver disease	17 (27.4)	22 (18.0)	13 (10.7)		
Other	5 (8.1)	4 (3.3)	2 (1.6)		
Max. tumor diameter (cm), M(IQR)	7.1 (5.5–8.9)	5.5 (4.1–7.3)	4.3 (3.0–5.9)	*F* = 35.21ᴬ	<0.001
Multifocal tumor (≥2), *n* (%)	33 (53.2)	65 (53.3)	40 (32.8)	χ^2^ = 10.846	0.004
Splenic length (cm)	15.48 ± 2.98	14.56 ± 2.71	13.05 ± 2.40	*F* = 21.367	<0.001
Total bilirubin (μmol/L), M(IQR)	74.5 (58.2–98.1)	35.2 (26.4–47.8)	16.8 (13.5–21.1)	*F* = 205.9ᴬ	<0.001
Albumin (g/L)	27.84 ± 4.15	32.11 ± 3.92	38.45 ± 3.89	*F* = 178.5	<0.001
INR, M(IQR)	1.94 (1.75–2.19)	1.45 (1.32–1.60)	1.10 (1.04–1.18)	*F* = 231.7ᴬ	<0.001
Creatinine (μmol/L), M(IQR)	115.4 (94.2–143.7)	81.3 (71.5–95.0)	70.1 (63.5–77.8)	*F* = 65.3ᴬ	<0.001
RDW (%)	16.91 ± 1.69	15.38 ± 1.47	13.91 ± 1.18	*F* = 94.8	<0.001
Platelet count (×10^9^/L)	65.44 ± 33.05	81.56 ± 40.12	105.23 ± 44.78	*F* = 19.25	<0.001
Child-Pugh class, *n* (%)				χ^2^ = 182.6	<0.001
Class A	4 (6.5)	61 (50.0)	118 (96.7)		
Class B	31 (50.0)	57 (46.7)	4 (3.3)		
Class C	27 (43.5)	4 (3.3)	0 (0.0)		
Ascites, *n* (%)	48 (77.4)	52 (42.6)	5 (4.1)	χ^2^ = 73.4	<0.001
Hepatic encephalopathy, *n* (%)	21 (33.9)	15 (12.3)	2 (1.6)	χ^2^ = 32.1	<0.001
cTACE/DEB-TACE [cTACE, *n*(%)]	41 (66.1)	88 (72.1)	102 (83.6)	χ^2^ = 7.12	0.028

### 3.3 Comparison of survival outcomes by Child-Pugh class

**Figure 4:**
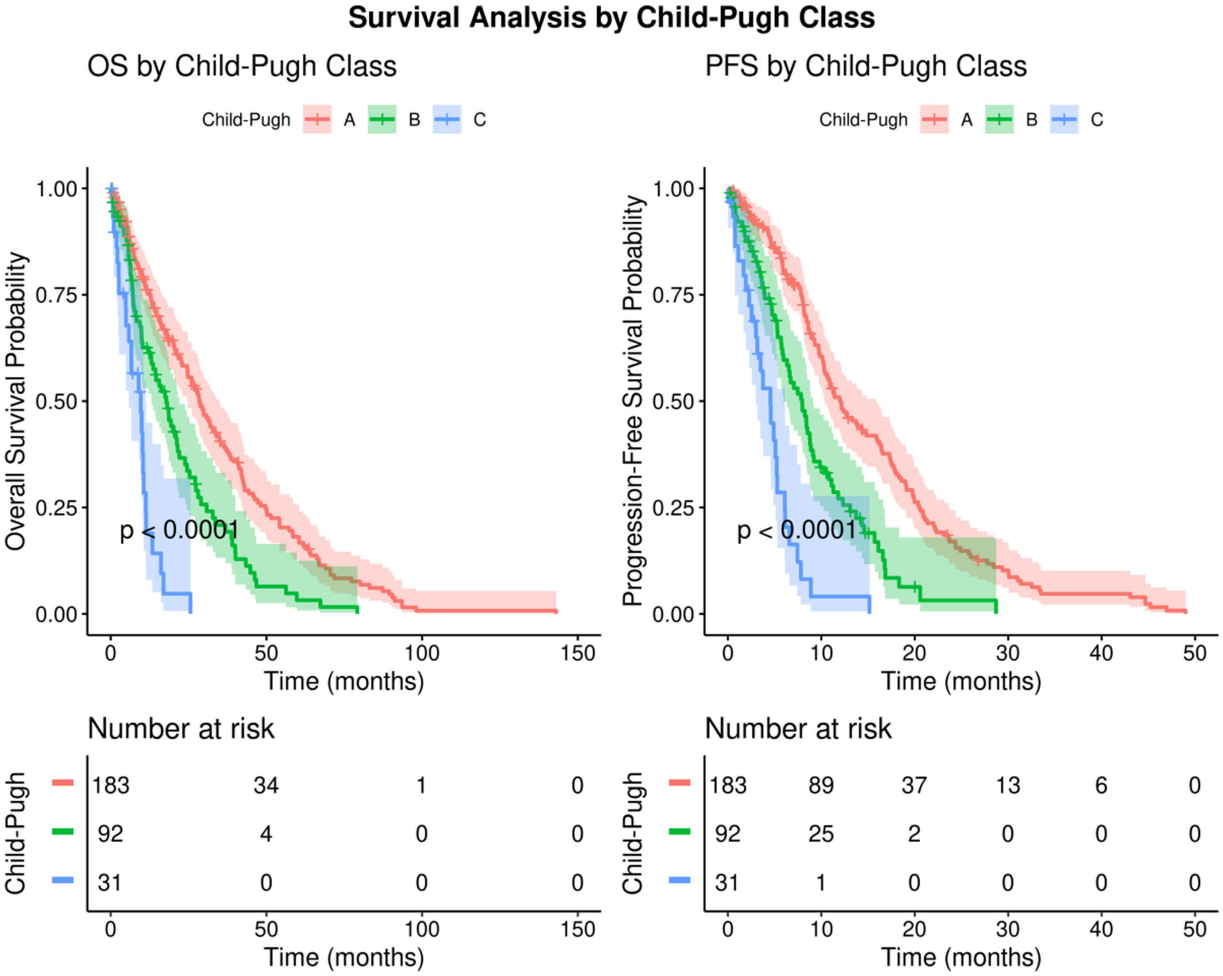
Comparison of survival outcomes by Child-Pugh class.

An analysis was conducted on the survival outcomes of different Child Pugh grades. The Log rank test showed that there were significant differences in overall survival (OS) and progression free survival (PFS) among the groups (*p* < 0.05). As the Child Pugh grading increases from A to C, there is a significant trend towards a shortened median OS and median PFS. Correspondingly, the 1-year and 2-year survival rates, as well as the 1-year and 6-month progression free rates, gradually decrease with the improvement of Child Pug grading (see Table 3; Figure 4).

**Table 3:** Comparison of survival outcomes by Child-Pugh class.

Survival indicator	Child-Pugh A (*n* = 183)	Child-Pugh B (*n* = 92)	Child-Pugh C (*n* = 31)	Log-rank test (χ^2^)	*p*-value
Overall survival (OS)				45.332	<0.001
Median OS, months (95% CI)	28.6 (24.3–32.9)	17.9 (14.5–21.3)	9.7 (6.8–12.6)		
1-year OS rate, % (95% CI)	75.6 (68.9–81.2)	62.6 (51.9–71.8)	19.0 (7.8–34.2)		
2-year OS rate, % (95% CI)	58.3 (50.6–65.3)	35.1 (25.3–45.1)	4.7 (0.8–14.5)		
Progression-free survival (PFS)				38.775	<0.001
Median PFS, months (95% CI)	12.1 (10.2–14.0)	8.0 (6.5–9.5)	4.5 (3.2–5.8)		
6-month PFS rate, % (95% CI)	80.5 (73.9–85.6)	59.7 (48.9–69.0)	28.5 (14.0–44.9)		

### 3.4 Comparison of survival outcomes by MELD score

**Figure 5:**
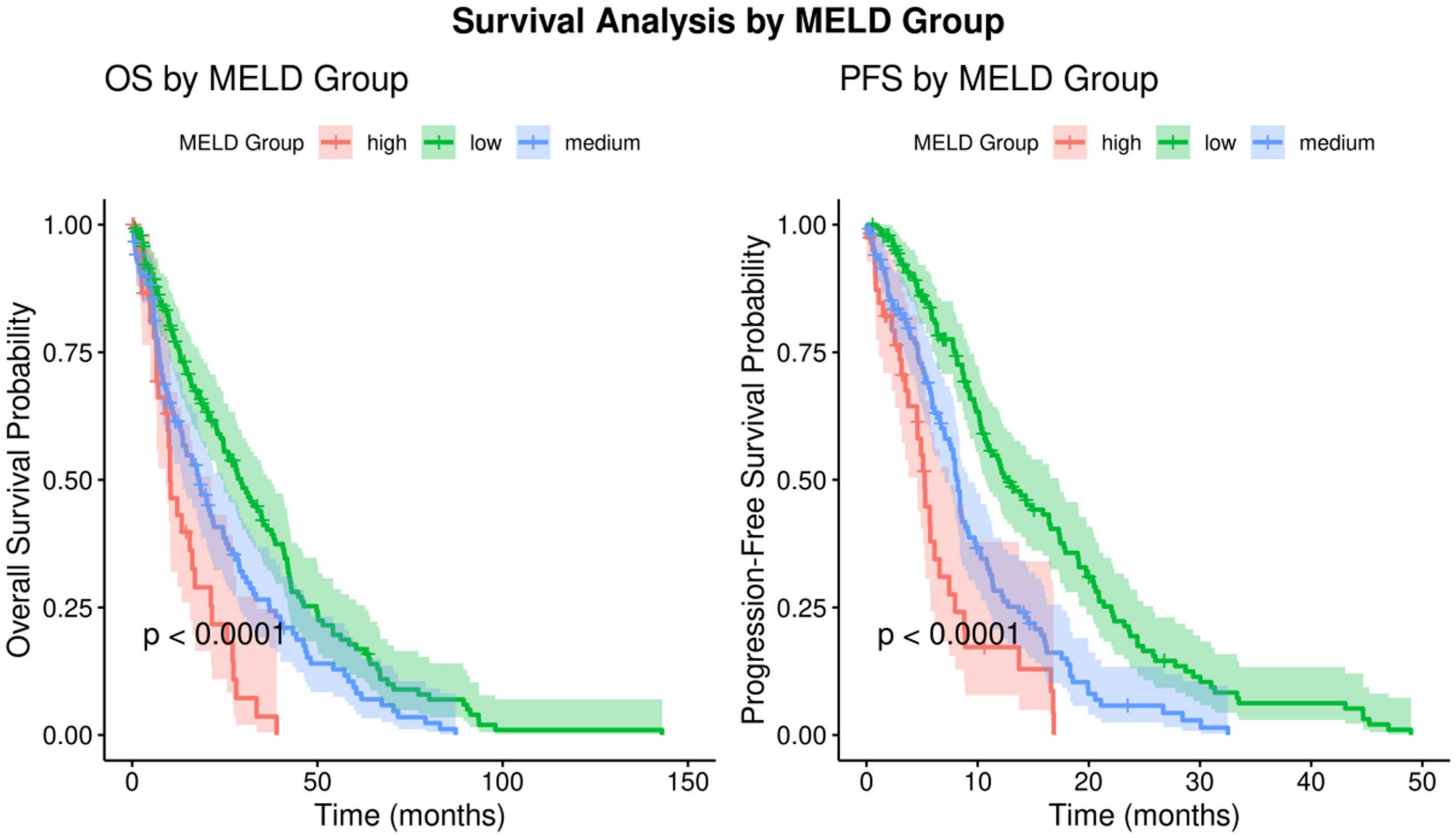
Comparison of survival outcomes by MELD score.

Compare the survival outcomes of patients with different MELD risk categories. Survival analysis showed that there were significant differences in OS and PFS among the risk groups statistically (*p* < 0.05). Specifically, as the MELD risk category increases from low to high, the median OS and median PFS gradually shorten. The corresponding 1-year and 2-year survival rates, as well as 1-year and 6-month progression free survival rates, have also shown a gradual downward trend (see Table 4; Figure 5).

**Table 4:** Comparison of survival outcomes by MELD score.

Survival Indicator	MELD<10 (low-risk, *n* = 122)	MELD 10–20 (Int.-risk, *n* = 122)	MELD>20 (high-risk, *n* = 62)	Log-rank test (χ^2^)	*p*-value
Overall survival (OS)				52.114	<0.001
Median OS, months (95% CI)	29.4 (25.1–33.7)	18.2 (15.3–21.1)	10.2 (7.9–12.5)		
1-year OS rate, % (95% CI)	76.3 (67.8–82.9)	61.4 (52.2–69.5)	46.4 (33.7–58.2)		
2-year OS rate, % (95% CI)	58.1 (48.8–66.4)	40.7 (31.8–49.5)	21.7 (12.3–33.0)		
Progression-Free Survival (PFS)				41.889	<0.001
Median PFS, months (95% CI)	12.7 (10.6–14.8)	8.1 (6.8–9.4)	5.2 (3.9–6.5)		
6-month PFS rate, % (95% CI)	81.5 (73.4–87.5)	64.1 (54.8–72.1)	37.9 (25.9–50.0)		
1-year PFS rate, % (95% CI)	53.1 (43.9–61.7)	28.3 (20.5–36.7)	17.2 (8.9–27.8)		

### 3.5 Comparison of predictive performance for 1-year survival: Child-Pugh vs. MELD scores

Use receiver operating characteristic (ROC) curve analysis to evaluate the predictive performance of a combination model of Child Pugh score, MELD score, and 1-year survival status. The results showed that the area under the curve (AUC) of MELD score was greater than that of Child Pugh score. Further DeLong tests showed that the predictive performance of MELD score was significantly better than that of Child Pugh score (*p* < 0.05). The predictive performance of the joint model is also significantly better than using Child Pugh score alone (*p* < 0.05). However, no statistically significant difference (*p* > 0.05) was observed between the predictive performance of the joint model and the MELD score used alone (see Tables 5, 6).

**Table 5:** Comparison of predictive performance for 1-year survival (ROC curve analysis).

Scoring System	AUC (95% CI)	Std. error	Optimal cut-off	Sensitivity (%)	Specificity (%)	Youden’s index
Child-Pugh score	0.723 (0.671–0.775)	0.027	>7 (Class B/C)	68.4	69.1	0.375
MELD score	0.781 (0.732–0.830)	0.025	>18	76.5	72.3	0.488
Combined model (Child-Pugh + MELD)	0.802 (0.755–0.849)	0.024	-	79.2	70.8	0.5

**Table 6:** Pairwise comparison tests (DeLong’s test).

Comparison	Z-value	*p*-value
MELD vs. Child-Pugh	3.124	0.002
Combined model vs. Child-Pugh	4.001	<0.001
Combined model vs. MELD	1.554	0.12

### 3.6 Multivariable cox regression analysis for independent prognostic factors

To determine the independent prognostic value of the Child-Pugh and MELD scores, we performed multivariable Cox regression analyses for OS and PFS, adjusting for age, etiology, maximum tumor diameter, multifocality, splenic length, and platelet count. Vascular invasion and distant metastasis were not included in any model because patients with these conditions were excluded from the cohort by design (Tables 7–10). In the multivariable model for OS, after adjusting for age, etiology, maximum tumor diameter, multifocality, splenic length, and platelet count, the MELD score (treated as a continuous variable) remained a significant independent predictor of mortality (HR = 1.08, 95% CI: 1.04–1.12, *p* < 0.001). In contrast, the Child-Pugh score (treated as a continuous variable) did not retain statistical significance in the final model (HR = 1.15, 95% CI: 0.98–1.35, *p* = 0.082). Other independent predictors of OS included maximum tumor diameter (HR = 1.21, 95% CI: 1.10–1.33, *p* < 0.001) and multifocality (HR = 1.68, 95% CI: 1.15–2.45, *p* = 0.007). For PFS, the MELD score (HR = 1.06, 95% CI: 1.02–1.10, *p* = 0.002), but not the Child-Pugh score (HR = 1.10, 95% CI: 0.94–1.29, *p* = 0.231), was an independent predictor of progression or death.

**Table 7:** Univariable cox regression analysis for overall survival (OS).

Variable	HR (95% CI)	*p*-value
MELD score (per 1-unit increase)	1.12 (1.08–1.16)	<0.001
Child-Pugh score (per 1-unit increase)	1.45 (1.29–1.63)	<0.001
Age (per 1-year increase)	1.02 (1.00–1.04)	0.045
Male (vs. female)	1.15 (0.82–1.61)	0.412
Etiology (non-HBV vs. HBV)	1.32 (0.98–1.78)	0.068
Max. tumor diameter (per 1-cm increase)	1.28 (1.18–1.39)	<0.001
Multifocality (≥2 vs. solitary)	1.95 (1.44–2.64)	<0.001
Splenic length (per 1-cm increase)	1.12 (1.06–1.18)	<0.001
Platelet count (per 10 × 10^9^/L increase)	0.95 (0.91–0.99)	0.012

**Table 8:** Multivariable cox regression analysis for overall survival (OS).

Variable	HR (95% CI)	*p*-value
MELD score (per 1-unit increase)	1.08 (1.04–1.12)	<0.001
Child-Pugh score (per 1-unit increase)	1.15 (0.98–1.35)	0.082
Max. tumor diameter (per 1-cm increase)	1.21 (1.10–1.33)	<0.001
Multifocality (≥2 vs. solitary)	1.68 (1.15–2.45)	0.007
Age (per 1-year increase)	1.01 (0.99–1.03)	0.312
Etiology (non-HBV vs. HBV)	1.18 (0.87–1.60)	0.287
Splenic length (per 1-cm increase)	1.05 (0.98–1.12)	0.154
Platelet count (per 10 × 10^9^/L increase)	0.97 (0.93–1.01)	0.142

HR, Hazard Ratio; CI, Confidence Interval; HBV, Hepatitis B Virus. The model was adjusted for all variables listed in the table.

**Table 9:** Univariable cox regression analysis for progression-free survival (PFS).

Variable	HR (95% CI)	*p*-value
MELD score (per 1-unit increase)	1.09 (1.05–1.13)	<0.001
Child-Pugh score (per 1-unit increase)	1.38 (1.23–1.55)	<0.001
Age (per 1-year increase)	1.01 (0.99–1.03)	0.312
Male (vs. female)	1.09 (0.79–1.50)	0.601
Etiology (non-HBV vs. HBV)	1.21 (0.91–1.61)	0.189
Max. tumor diameter (per 1-cm increase)	1.22 (1.13–1.32)	<0.001
Multifocality (≥2 vs. solitary)	1.78 (1.34–2.36)	<0.001
Splenic length (per 1-cm increase)	1.09 (1.04–1.14)	<0.001
Platelet count (per 10 × 10^9^/L increase)	0.96 (0.92–1.00)	0.048

**Table 10:** Multivariable cox regression analysis for progression-free survival (PFS).

Variable	HR (95% CI)	*p*-value
MELD score (per 1-unit increase)	1.06 (1.02–1.10)	0.002
Child-Pugh score (per 1-unit increase)	1.10 (0.94–1.29)	0.231
Max. tumor diameter (per 1-cm increase)	1.15 (1.05–1.26)	0.002
Multifocality (≥2 vs. solitary)	1.59 (1.12–2.26)	0.009
Age (per 1-year increase)	1.00 (0.98–1.02)	0.678
Etiology (non-HBV vs. HBV)	1.12 (0.84–1.49)	0.441
Splenic length (per 1-cm increase)	1.03 (0.97–1.09)	0.323
Platelet count (per 10 × 10^9^/L increase)	0.98 (0.94–1.02)	0.311

### 3.7 Subgroup analysis according to splenomegaly severity

To evaluate the impact of splenomegaly severity on prognosis and on the predictive performance of the scoring systems, patients were stratified into three groups based on splenic length tertiles: mild splenomegaly (splenic length ≤13 cm, *n* = 102), moderate splenomegaly (13–16 cm, *n* = 124), and severe splenomegaly (>16 cm, *n* = 80).

The Kaplan–Meier analysis showed that as the severity of splenomegaly increased, both OS and PFS significantly decreased. The median OS for the mild group was 25.3 months (95% CI: 21.1–29.5), for the moderate group it was 17.8 months (95% CI: 14.6–21.0), and for the severe group it was 11.2 months, 95% CI: 8.5–13.9 (log-rank χ^2^ = 31.447, *p* < 0.001). The median progression-free survival was 11.5 months (95% CI: 9.8–13.2), 8.2 months (95% CI: 6.7–9.7), and 5.4 months (95% CI: 4.1–6.7) respectively (log-rank χ^2^ = 28.113, *p* < 0.001).

ROC analysis was performed separately for 1-year survival prediction in each splenomegaly severity group. At all levels, the MELD score consistently outperformed the Child-Pugh score, with the greatest difference observed in the severe splenomegaly group (AUC MELD 0.789 vs. Child-Pugh 0.711, DeLong test Z = 2.456, *p* = 0.014).

In the multivariate Cox model of OS, including splenomegaly severity (as a categorical variable) and MELD score, Child-Pugh score, and other confounding factors, severe splenomegaly (compared to mild splenomegaly) remained an independent predictor of mortality (HR = 1.67, 95% CI: 1.21–2.30, *p* = 0.002), and the MELD score retained its independent prognostic value (HR = 1.06, 95% CI = 1.02–1.10, *p* = 0.003). The Child-Pugh score was not significant in this model (HR = 1.12, 95% CI: 0.95–1.32, *p* = 0.178). These results indicate that the severity of splenomegaly provides additional independent prognostic information beyond the MELD score.

### 3.8 Subgroup analysis based on TACE mode (cTACE vs. DEB-TACE)

To evaluate whether the prognostic performance of Child-Pugh and MELD scores varies by TACE mode, we conducted separate survival analyses and ROC curve comparisons in the cTACE (*n* = 273) and DEB-TACE (*n* = 33) subgroups. Due to the small number of DEB-TACE patients, the analysis was descriptive. In the cTACE subgroup, the predictive performance of MELD score for 1-year survival rate was significantly better compared to Child-Pugh score (AUC: 0.777 vs. 0.718, DeLong test *p* = 0.008). In the DEB-TACE subgroup, the point estimates tended to favor MELD (AUC: 0.798 vs. 0.741), but due to the limited sample size, the difference was not statistically significant (*p* = 0.312). In multivariate analysis, there was no significant interaction between the prognostic impact of TACE pattern and MELD score (P for interaction = 0.421). These results indicate that the superiority of the MELD score is consistent across TACE modalities despite insufficient DEB-TACE subgroup analysis.

### 3.9 Model discrimination performance (C index) of OS and PFS

**Figure 6:**
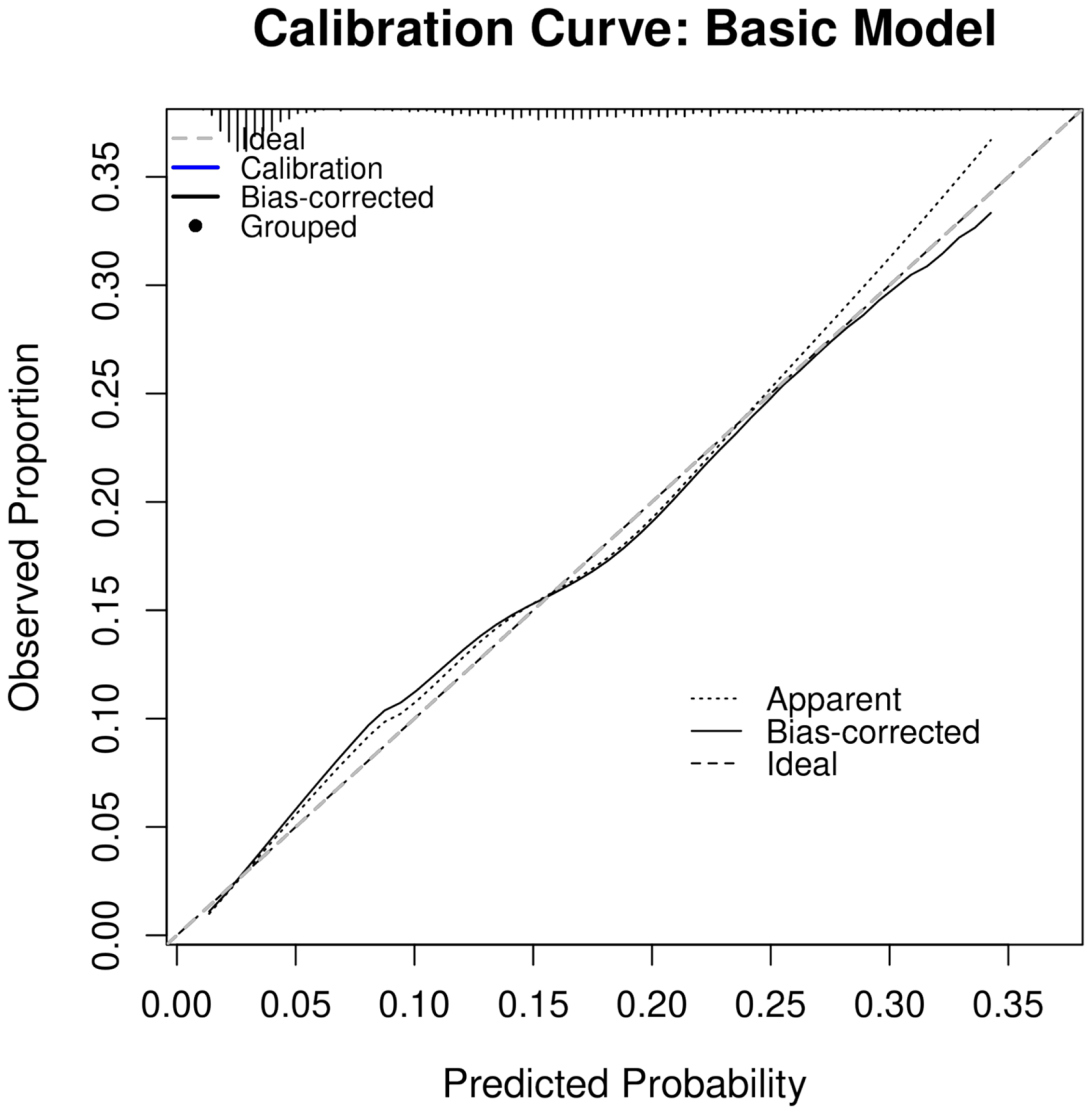
Calibration curves.

**Figure 7:**
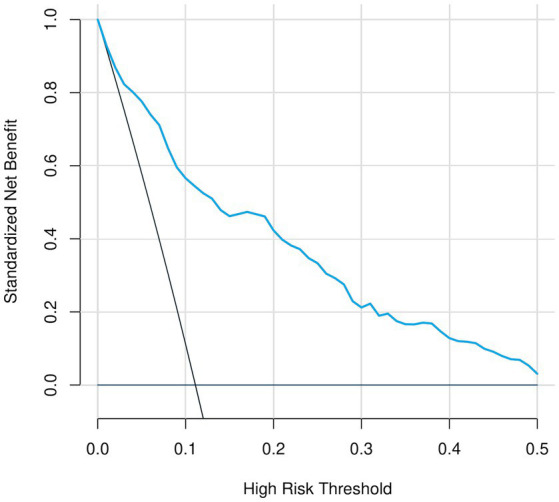
Decision curve analysis.

The C-index (concordance index) was calculated to compare the discriminative ability of the Child-Pugh score, MELD score, and the combined model for predicting 1-, 2-, and 3-year OS and PFS. Table 11 summarizes the results. The MELD score consistently showed higher C-index values than the Child-Pugh score at all time points, and the combined model did not show significant improvement over the individual MELD scores. The calibration curve and decision curve validated the good predictive performance of the model (Figures 6, 7).

**Table 11:** C-index (95% CI) for Child-Pugh, MELD, and combined models.

Outcome	Child-Pugh score	MELD score	Combined model
1-year OS	0.712 (0.668–0.756)	0.768 (0.726–0.810)	0.789 (0.748–0.830)
2-year OS	0.698 (0.651–0.745)	0.751 (0.706–0.796)	0.770 (0.726–0.814)
3-year OS	0.685 (0.634–0.736)	0.739 (0.690–0.788)	0.755 (0.707–0.803)
PFS	0.674 (0.628–0.720)	0.721 (0.677–0.765)	0.734 (0.691–0.777)

## 4 Discussion

The aim of this study is to evaluate and compare the predictive value of Child Pugh and Model of End Stage Liver Disease (MELD) scores for survival in hepatocellular carcinoma patients with splenomegaly after arterial chemoembolization in a real-world context. The analysis results show that both scoring systems can effectively distinguish the prognostic risk of patients, but the MELD score shows significantly better discriminatory efficacy in predicting one-year postoperative survival (16). This core finding suggests that for this specific population, MELD scores based on objective laboratory indicators may provide more accurate prognostic information than Child Pugh grading that includes subjective clinical evaluations.

According to the comparison of baseline characteristics based on Child Pugh grading, as liver function deteriorates from grade A to grade C, the tumor burden of patients shows an increase in maximum diameter and proportion of multiple tumors, while the length of the spleen also significantly increases. This phenomenon may be attributed to the pathophysiological relationship between liver dysfunction and portal hypertension; Progressive exacerbation of portal hypertension is not only a direct driving factor for splenomegaly, but its accompanying systemic inflammatory state and hemodynamic changes may also create a favorable microenvironment for tumor growth and progression (17). In addition, indicators such as the width of red blood cell distribution that reflect inflammation and nutritional status increase with the deterioration of Child Pugh grade, further outlining the complex interplay between liver disease severity, systemic status, and tumor invasiveness.

The baseline analysis based on MELD score grouping showed a similar trend, but there was a significant overlap between the grouping and Child Pugh grading, especially in the medium to high risk group. This indirectly confirms that the MELD score, by integrating continuous variables such as bilirubin, international standardized ratio of coagulation, and creatinine, can more sensitively quantify the continuous spectrum of liver synthesis failure and potential renal dysfunction (18). In contrast, although ascites and hepatic encephalopathy assessment in Child Pugh grading are clinically important, they are susceptible to diuretic treatment, subjective interpretation, and intermittent fluctuations, which may lead to lag or bias in the staged judgment of the severity of liver disease in the same patient. Therefore, MELD score may be able to identify subgroups of patients who are classified similarly in Child Pugh grading but have actually poorer intrinsic physiological reserves.

Survival analysis shows that, whether based on Child Pugh grading or MELD score, poorer grades or higher scores are closely associated with shorter overall survival and progression free survival. It is worth noting that the median survival of Child Pugh B or MELD risk group patients in this cohort is shorter than data reported in previous HCC studies that did not specifically screen for splenomegaly (19). This difference reinforces the adverse prognostic significance of splenomegaly as a key biomarker of portal hypertension, which may worsen survival outcomes by exacerbating blood cell depletion associated with splenic hyperfunction, promoting systemic inflammatory response, and limiting subsequent treatment tolerance at multiple levels (20).

The most crucial analysis lies in the direct comparison of predictive performance, where the area under the MELD score curve is significantly better than the Child Pugh score. The higher sensitivity of MELD score means that it can more effectively screen patients with extremely high risk of death within one year after surgery (21). Its advantage may stem from the inclusion of creatinine, which directly reflects kidney function; In patients with advanced liver disease accompanied by significant portal hypertension, the incidence of functional kidney injury (such as hepatorenal syndrome) is high and strongly correlated with mortality, which is not included in the Child Pugh scoring system (22). Although the joint model achieved the highest area under the curve, it did not significantly surpass the MELD score, indicating that under the existing parameter framework, MELD score already covers the main prognostic information, and the incremental benefits of increasing Child Pugh score are limited (23, 24).

This study has some limitations. Firstly, as this is a single-center retrospective analysis, the general applicability of our conclusions is inherently limited. Although we conducted internal validation through multivariate adjustment, thereby enhancing the reliability of the research results, it is crucial to conduct external validation from different independent institutions and different cohorts in different regions before these results can be widely applied in clinical practice. In the future, it is necessary to carry out multi-center prospective studies to confirm the superiority of the independent prognostic value of MELD score in this specific patient population. Secondly, the assessment of splenomegaly mainly relies on length measurement, and does not include more accurate indicators such as spleen volume or spleen hardness. This may not fully capture the dose–response relationship between spleen pathology and prognosis. Future research may focus on developing new prognostic models that combine spleen-specific parameters (such as the platelet count/spleen diameter ratio) with the MELD score, aiming to provide more personalized risk stratification tools for HCC patients with splenomegaly. Third, we acknowledge that treatment-related heterogeneity may affect outcomes. Although all TACE procedures were performed at a single center according to standardized protocols, there were differences in the selection of chemotherapy drugs, embolization materials, and between conventional TACE and drug-eluting bead TACE. The proportion of DEB-TACE was relatively small (10.8%), limiting the statistical power of subgroup analysis. Although we did not find a significant interaction between TACE modality and the prognostic performance of the MELD score, this finding should be interpreted with caution. The decision to repeat TACE and subsequent treatments (such as ablation and systemic therapy) after progression was not standardized, which may affect post-progression survival. Due to the complexity and non-random nature of these real-world treatment decisions, we were unable to fully adjust for these factors in our analysis. When interpreting survival outcomes, this heterogeneity should be taken into account. Fourth, although we provided detailed rationale for including 31 Child-Pugh C patients, TACE is typically contraindicated in this population according to international guidelines. These patients were highly selected through strict MDT supervision and enhanced perioperative management in a tertiary center. Therefore, our findings in Child-Pugh C patients may not be applicable to other settings, and without similar strict selection criteria and perioperative support, TACE should not be routinely performed in Child-Pugh C patients.

In conclusion, in a single-center cohort of HCC patients with splenomegaly undergoing TACE, the MELD score had better predictive performance for short-term survival compared with the Child-Pugh classification. These findings suggest that the MELD score may be more useful in preoperative risk stratification and prognostic assessment for this specific patient population and may help identify high-risk individuals to inform treatment decisions and follow-up strategies. However, external validation in larger multicenter cohorts is needed before these findings can be generalized to wider clinical practice.

## Data Availability

The datasets presented in this article are not readily available because access to the de-identified dataset may be granted upon reasonable request and subject to approval by the Hospital's Ethics Committee. Researchers who wish to request the data should contact the corresponding author. Any data sharing will require a formal data use agreement to ensure compliance with ethical standards and institutional policies. Requests to access the datasets should be directed to Dong Lu; ludong@ahmu.edu.cn.
